# Effectiveness and safety of cyclophosphamide or tacrolimus therapy for idiopathic membranous nephropathy

**DOI:** 10.1080/0886022X.2019.1637758

**Published:** 2019-07-29

**Authors:** Honghong Zou, Fang Jiang, Gaosi Xu

**Affiliations:** a Department of Nephrology, the Second Affiliated Hospital of Nanchang University, Nanchang, China;; b Department of Nephrology, People's Hospital of Xinyu City, Xinyu, China

**Keywords:** Idiopathic membranous nephropathy, tacrolimus, cyclophosphamide, retrospective study, effectiveness

## Abstract

**Background:** Guidelines recommend combined therapy of glucocorticoid and cyclophosphamide (CYC) for patients with idiopathic membranous nephropathy (IMN), while it is associated with severe adverse effects. We conducted a retrospective study to evaluate the effectiveness and safety of glucocorticoid plus tacrolimus (TAC) for IMN.

**Methods:** Two hundred and three kidney-biopsy-proven IMN patients were enrolled in this study. One group (*n* = 142) received glucocorticoid combined with intravenous CYC (750 mg/m^2^ body surface) and the other group (*n* = 61) received glucocorticoid combined with oral TAC (target blood concentration of 4–8 ng/mL). The primary outcomes were achievement of remission and incidence of adverse events. The secondary end points included relapse rates, 24 h urinary protein (UP), serum albumin, serum creatinine and estimated glomerular filtration rate.

**Results:** Over the 18-month observation period, the study suggested that the remission rates at the first 3 months were significantly higher in TAC group than in CYC group (72.1% versus 54.9%, *p* < .05). Although the cumulative incidence of serious and non-serious adverse events was not different significantly between the two groups, the incidence after first 3 months was lower in TAC group. 24hUP and serum albumin improved in TAC group more than the CYC group (*p* < .05) over the observed period.

**Conclusion:** Because of its short-term effectiveness and long-term safety profile, glucocorticoid plus TAC might be a better option for IMN.

## Introduction

Idiopathic membranous nephropathy (IMN) is one of the leading causes of nephrotic syndrome (NS) in adults [1]. Current guidelines recommend glucocorticoid plus cyclophosphamide (CYC) as the initial therapy for patients with IMN [2]. Although this combined regimen has demonstrated a good effectiveness in remission, is associated with severe side effects [3,4]. Considering the advanced age of the majority of IMN patients, numerous adverse effects of these aggressive regimen are an important concern. Furthermore, a considerable number of IMN patients achieve spontaneous remission during the course of disease. According to these caveats, some physicians reluctantly implement the combined therapy of glucocorticoid and CYC to those patients with more unfavorable prognostic markers [5,6].

As one of the calcineurin inhibitors, an RCT study showed that a majority of patients who received tacrolimus (TAC) monotherapy experienced remission with a significant reduction in the risk for deteriorating renal function in IMN [7]. Besides, some previously reported studies indicated that the combination of TAC and glucocorticoid was as effective as the combination of CYC and glucocorticoid for IMN patients in achieving remission of severe proteinuria, and a higher incidence of remission was found in the short term in combination of TAC and glucocorticoid [8–10]. However, most studies had a small sample size and short-term follow-up, and few studies have been conducted to compare the safety profiles in two treatment protocols [8–10]. Thus, we performed a retrospective study. Our working hypothesis was that the combined therapy of TAC and glucocorticoid was more effective and safer than CYC combined glucocorticoid for IMN patients.

## Methods

### Patients

The retrospective study was conducted from 1 March 2011 to 28 February 2017. All the revealed patients who were presenting to the Department of Nephrology, the Second Affiliated Hospital of Nanchang University, Jiangxi Province, China would be screened out according to the enrollment and exclusion criteria. The enrollment criteria were as follows: (1) IMN (stage I–IV) proven by renal biopsy; (2) aged between 16 and 70 years old; (3) 24 h (h) urinary protein (UP)>3.5 g, serum albumin < 30 g/L, edema, and/or hyperlipidemia; (4) initial serum creatinine (Scr)<176 μmol/L. The exclusion criteria were as following: (1) malignancy, diabetes mellitus, systemic lupus erythematosus, or any other systemic disease known to be associated with secondary IMN, infections (including hepatitis B and C virus and HIV), pregnancy or lactating; (2) treatment with glucocorticoids or immunosuppressive therapy within 6-month period before enrollment; (3) life-threatening complications such as heart failure or severe infections. The study was approved by the Ethics Committee of the Second Affiliated Hospital of Nanchang University (IRB approval number #20110118009) and conducted according to the Declaration of Helsinki. All participants signed the informed consent for releasing their data obtained during routine clinical care before the procedure.

### Treatment protocol

A total of 330 patients who received glucocorticoid with either CYC or TAC were enrolled in. Eventually, 203 patients were accessed for eligibility. The patient preference dictated which of the drugs each person received during routine clinical care. The eligible patients treated with CYC and glucocorticoid were considered the control group (*n* = 142) and those treated with TAC and glucocorticoid (*n* = 61) were considered the treatment group according to the medication records for an 18-month follow-up. Patients in the TAC group were initially received TAC at a dosage of 0.05 mg/kg per day (no more than 0.15 mg/kg per day), which was divided into two equal doses at 12 h intervals. The dosage was adjusted according to the whole blood concentration of a target of 4–8 ng/mL. TAC was continued for 12 months and then gradually tapered off (25% reduction of the dose) for the next 6 months. Patients in the TAC group received oral glucocorticoid 0.5 mg/kg per day (no less than 30 mg/day) for 8 weeks, and then gradually tapered off (5 mg reduction for every 4 weeks) until 10 mg/day. Subsequently, the dose was reduced again slowly until complete withdrawal at the end of 12 months.

In the CYC group, CYC was administered intravenously at 750 mg/m^2^ body surface once every 4 weeks for 6–12 moths (cumulative dosage no more than 8 g). Patients were given an oral prednisone dose of 0.8–1.0 mg/kg per day (no more than 60 mg/day) for 8 weeks, and then gradually reduced the dosage by 5 mg per 4 week to 10 mg/day. Subsequently, the dose was reduced again slowly until complete withdrawal at the end of 12 months.

### Follow-up and outcomes

Follow-up participants were scheduled at 3, 6, 12 and 18 months after the initiation of the immunosuppressive therapy described above. We predefined potential baseline indexes including sex, age, standard complete blood count, serum albumin, 24 h UP, estimated glomerular filtration rate (eGFR), Scr, aminotransferase, glucose, total cholesterol and triglycerides, and trough levels of TAC. In addition, the use of angiotensin-converting enzyme inhibitor (ACEI)/angiotensin receptor blocker (ARB) and statin was observed in this patient population.

The primary outcomes were as following: (1) Time to complete remission (CR) defined as 24 h UP < 0.3 g with normal Scr concentration; (2) Time to partial remission (PR) defined as 24 h UP at 0.3–3.5 g and 50% lower than baseline 24 h UP with a stable Scr concentration; (3) Time to first serious adverse event defined as any untoward medical incident: reaching death, permanent or significant disability, is a congenital abnormality or birth defect in offspring, life-threatening illness, require in-patient hospitalization or prolongation thereof; (4) Time to first non-serious adverse event defined as any adverse event not classified as serious.

Secondary outcomes were as following: (1) Time to no response (NR) defined as a decrease in 24 h UP less than 50% and/or 24 h UP > 3.5 g with a normal Scr concentration; (2) Time to relapses defined new NS after CR or PR, without remission after two weeks; (3) The evolution of 24 h UP, serum albumin, eGFR and renal survival. The CKD-EPI_2009Scr_ was used to calculated eGFR. Renal survival was defined as on the basis of a 50% increase in baseline Scr concentrations. TAC dosage were reduced by 25% every 2 weeks in the presence of a 50% Scr increase in the TAC group. Once the increasing of Scr persisted 50% of baseline values for 2–4 weeks after 75% reduction of TAC doses, definition of end point was established.

### Statistical analysis

We expressed baseline characteristics as frequencies and proportions for categorical variables, mean ± SD for normally distributed variables. We compared baseline characteristics of CYC and TAC treated patients by using Chi-square test and *t*-test. Cumulative incidences were compared with a log rank test. Differences of quantitative parameters between the two groups were evaluated by *t-*test or non-parametric tests. Differences of qualitative results were compared by using Chi-square test or fisher’s exact tests. A two-sided *p* values<.05 was considered statistically significant. Statistical analysis was performed with Graph Pad Prism (version 7.0) and SPSS (version 25.0).

## Results

### Characteristics of study population

**Figure 1.**
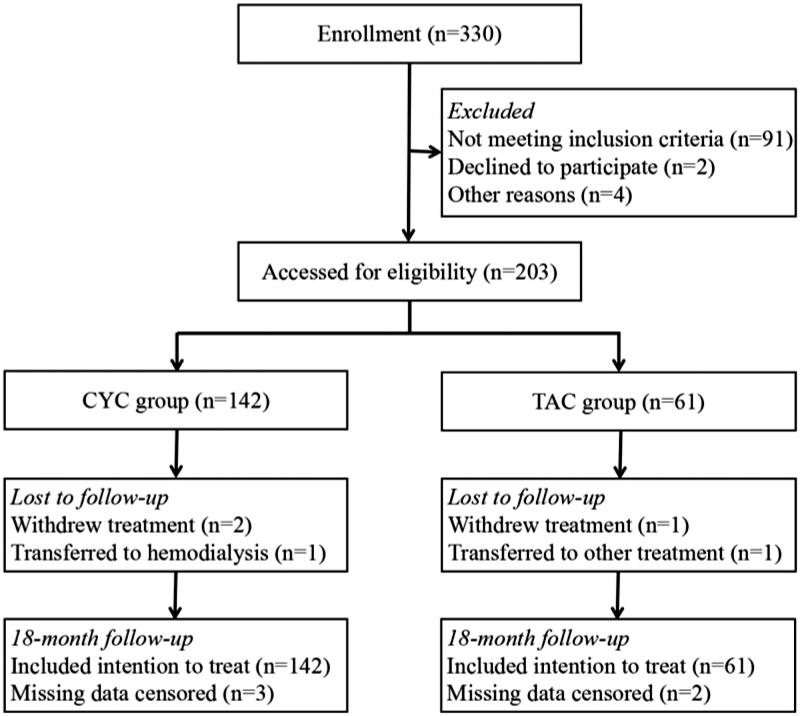
Flow diagram for inclusion of participants. CYC: cyclophosphamide; TAC: tacrolimus.

A flow chart of the patients’ selection is shown in Figure 1. Preliminary screening enrolled 330 patients and 203 subjects were finally included in the present study. That 5 cases dropped out was attributing to their inability for regular follow-up. All of the 203 patients were assigned to the CYC group (*n* = 142) or to the TAC group (*n* = 61). At baseline, patient characteristics were almost similar between the two cohorts (Table 1). 24 h UP, serum albumin, eGFR and Scr levels, total cholesterol and serum triglyceride etc. were comparable between the two groups. There was a significant difference in age distribution where the patients in the TAC group were younger.

**Table 1.** Baseline characteristics of patients with idiopathic membranous nephropathy and treated with cyclophosphamide or tacrolimus regimen.

Characteristic	CYC group (*n* = 142)	TAC group (*n* = 61)	*p* value
Men, %	58.5	55.7	.72
Age, year	46.84 ± 11.67	40.46 ± 17.16	.01
Systolic BP, mmHg	126.75 ± 18.26	127.44 ± 23.48	.82
Diastolic BP, mmHg	80.76 ± 12.45	80.43 ± 12.71	.86
24h UP, g/24h	8.68 ± 4.78	8.65 ± 5.19	.97
Serum albumin, g/L	24.78 ± 4.85	23.75 ± 5.15	.18
Scr, μmol/L	74.43 ± 25.80	79.25 ± 31.17	.25
eGFR, mL/min/1.73m^2^	97.75 ± 20.52	97.53 ± 26.16	.95
Total cholesterol, mmol/L	7.98 ± 2.32	8.52 ± 2.77	.15
Serum triglyceride, mmol/L	2.84 ± 1.92	2.48 ± 1.15	.10
ACEI/ARB use, %	40.8	34.4	.39
Statin use, %	30.3	26.2	.56

Data are presented as proportions (%), or means ± SD. *p* values: CYC group *vs* TAC group (Chi-square test or *t*-test). CYC: cyclophosphamide; TAC: tacrolimus; BP: blood pressure; h: hour; UP: urinary protein; eGFR: estimated glomerular filtration rate; Scr: serum creatinine; ACEI: angiotensin-converting enzyme inhibitor; ARB: angiotensin receptor blocker.

### Effectiveness outcomes

As shown in Table 2, a significantly higher rate of remission (CR + PR) was observed in the TAC group in the first 3 months of follow-up (72.1% versus (*vs*) 54.9%, *p* = .02). However, no significant difference was found between the two groups after the first 3 months of follow-up. The proportion of remissions in TAC group and CYC group was 73.8% *vs* 78.9%, respectively, by 6 months (*p* = .43), 90.2% *vs* 92.3% by 12 months (*p* = .62), and 91.8% *vs* 93.7% by 18 months (*p* = .63). At the follow-up visit of 18 months, NR was seen in 2 TAC patients and 3 CYC patients. 3 of 61 TAC patients (4.9%) had a relapse, whereas 5 of 142 CYC patients (3.5%) had a relapse. Furthermore, a single one CYC patient progressed to renal failure and underwent regular hemodialysis.

**Table 2.** Remission rates in idiopathic membranous nephropathy patients treated with cyclophosphamide or tacrolimus.

Group	Month			
	3	6	12	18
CYC group (*n* = 142)				
CR	31	36	80	100
PR	47	76	51	33
NR	64	28	7	3
Relapse	0	1	3	5
Renal Failure	0	1	1	1
Remission (CR + PR)	78	112	131	133
Remission rate (%)	54.9%	78.9%	92.3%	93.7%
TAC group (*n* = 61)				
CR	15	19	30	34
PR	29	26	25	22
NR	17	16	4	2
Relapse	0	0	2	3
Renal Failure	0	0	0	0
Remission (CR + PR)	44	45	55	56
Remission rate (%)	72.1%	73.8%	90.2%	91.8%
*p* value	.02	.43	.62	.63

*p* values: CYC group *vs* TAC group (Chi-square test). CYC: cyclophosphamide; TAC: tacrolimus; CR: complete remission; PR: partial remission; NR: non-responder.

### Safety outcomes

**Figure 2.**
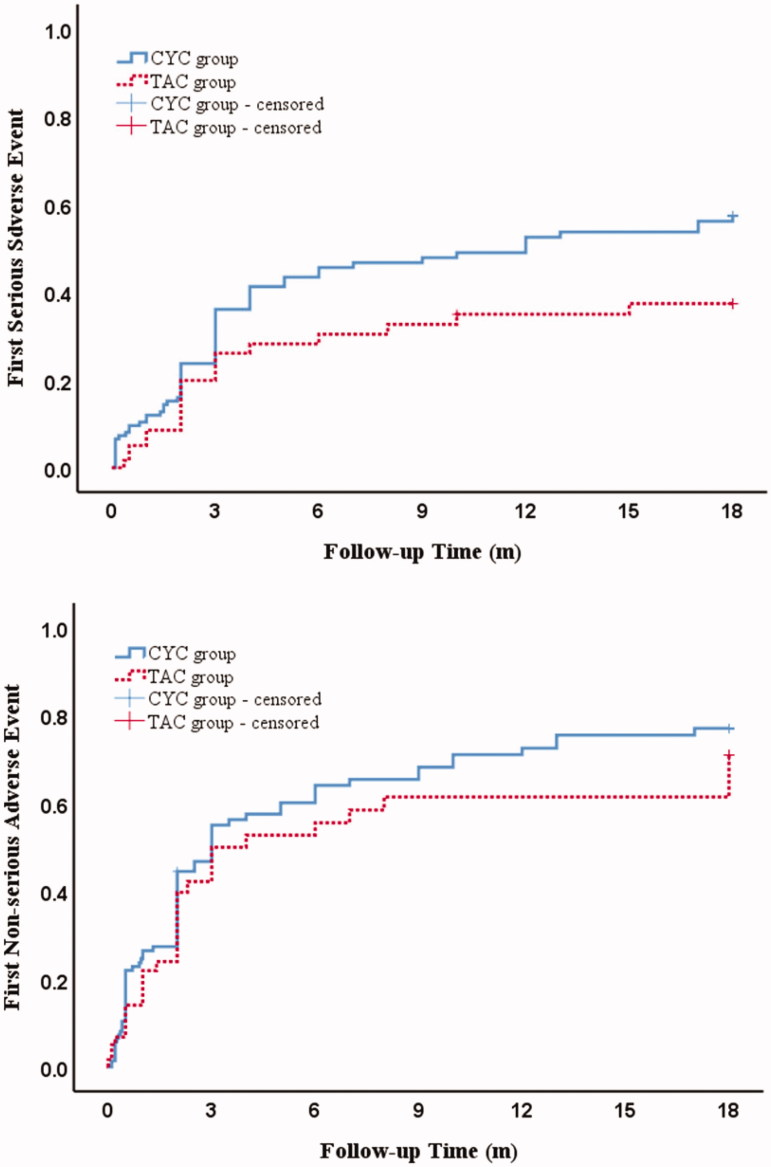
Cumulative incidence curves for safety outcomes in idiopathic membranous nephropathy patients treated with cyclophosphamide compared to tacrolimus. Cumulative incidence curves for (A) time to the first serious adverse event; or (B) time to the first non-serious adverse event. (A) and (B): *p* > .05. CYC group vs TAC group (log-rank test). CYC: cyclophosphamide; TAC: tacrolimus.

Over the 18-month follow-up, 62 records of any first serious adverse event were observed in the CYC group, whereas 19 records were observed in the TAC group (Tables 3 and 4). The cumulative incidence of first serious events was 56% in the CYC group, 38% in the TAC group (Figure 2). Besides, we also observed 76 records of any first non-serious adverse events in the CYC group and 31 records in the TAC group. The cumulative incidence of first non-serious events was 77% in the CYC group, 70% in the TAC group. It is obvious in Figure 2 that the cumulative incidence of first serious and non-serious adverse events was lower in the TAC group after the first 3 months follow-up. Nonetheless, the cumulative incidence curves showed an insignificance between two groups in serious adverse event (*p* = .11), as well as the first non-serious adverse event (*p* = .63). Overall, the hazards of the events considered separately were probably lower in the TAC group than the CYC group.

**Table 3.** Idiopathic membranous nephropathy patients with serious adverse events throughout the whole observation period according to treatment group.

Type of event	Total No.		Likely/Possibly Related		Unrelated		*p* value
	CYC group	TAC group	CYC group	TAC group	CYC group	TAC group	
Myelotoxicity	7	1	7	1	0	0	.58
Pancytopenia	1	0	1	0	0	0	1.00
Anemia	5	0	5	0	0	0	.58
Thrombocytopenia	1	1	1	1	0	0	.86
Cardiovascular and cerebrovascular events	3	1	0	0	3	1	1.00
Thromboembolic events	2	0	2	0	0	0	1.00
Pulmonary thromboembolism	1	0	1	0	0	0	1.00
Deep venous thrombosis	1	0	1	0	0	0	1.00
Infections	41	8	37	7	4	1	.39
Respiratory failure	2	1	2	1	0	0	1.00
Pneumonia	19	3	19	3	0	0	.59
Urinary tract	5	1	5	1	0	0	1.00
Other/unspecified	15	3	11	2	4	1	.95
Gastrointestinal symptoms	4	0	4	0	0	0	.58
New-onset diabetes mellitus	15	7	15	7	0	0	.12
Tuberculosis	2	0	2	0	0	0	1.00
Osteonecrosis	3	1	3	1	0	0	1.00
Other events	11	4	10	3	1	1	.73
Total no. of serious adverse events*	88	22	80	19	8	3	.81
Patients with first serious adverse events	62	19	58	16	4	3	.42

*One patient may have more than one event. *p* values: CYC group *vs* TAC group (Chi-square test). CYC: cyclophosphamide; TAC: tacrolimus.

**Table 4.** Idiopathic membranous nephropathy patients with non-serious adverse events throughout the whole observation period according to treatment group.

Type of event	Total No.		Likely/Possibly Related		Unrelated		*p* value
	CYC group	TAC group	CYC group	TAC group	CYC group	TAC group	
Infections	43	20	43	20	0	0	0.01
Respiratory tract	40	17	40	17	0	0	0.04
Epifolliculitis	3	3	3	3	0	0	0.20
Herpes zoster	3	1	3	1	0	0	1.00
Impaired glucose tolerance	12	1	12	1	0	0	0.40
Minor gastrointestinal symptoms	18	5	17	5	1	0	0.90
Hepatotoxicity	32	8	32	8	0	0	0.89
New-onset tachycardia	14	2	14	2	0	0	0.60
Minor cardiovascular disease	5	0	5	0	0	0	0.59
Muscular soreness	3	0	2	0	1	0	1.00
Limbs discomfort	6	0	6	0	0	0	0.35
Other events	9	1	8	1	1	0	0.64
Total no. of non-serious adverse events*	145	38	142	38	3	0	1.00
Patients with first non-serious adverse events	76	31	74	31	2	0	1.00

*One patient may have more than one event. *p* values: CYC group *vs* TAC group (Chi-square test). CYC: cyclophosphamide; TAC: tacrolimus

Considering a patient may have more than one event, we totally observed 233 adverse events (88 serious and 145 non-serious) in the CYC group and 60 adverse events (22 serious and 38 non-serious) in the TAC group during the whole follow-up period. Lists of the adverse events observed in two groups were presented in Tables 3 and 4. Among the whole adverse events reported in the two groups, almost half of the events were related to infection in both groups. The incidence of new-onset diabetes mellitus and impaired glucose tolerance accounted for a large proportion of the whole serious/non-serious adverse events. It might be possibly related to glucocorticoid exposure during the course of immunosuppression. Glucocorticoids dosage differs between the two treatment groups so higher rates of diabetes mellitus and impaired glucose tolerance are not unexpected in the CYC group. In addition, the incidence of hepatotoxicity, minor gastrointestinal symptoms and new-onset tachycardia also accounted for a large proportion of the whole non-serious adverse events, respectively.

Among the serious adverse events reported in the two groups, two patients in the CYC group and a single of the TAC group had respiratory failure caused by severe pneumonia. One patient in the CYC group died from respiratory failure after two months of the immunosuppressive therapy. Two patients in the CYC group were subjected from embolism. One of the patients was suffered from pulmonary embolism, while the other suffered from multiple venous embolization which included pulmonary embolism, renal venous thrombosis and inferior vena cava embolism. One fatal cardiovascular event was perhaps unrelated to the exposure of TAC and glucocorticoid, because he was observed with a previous history of cardiovascular disease. Although TAC is known to increase the cardiovascular risk, we do not have evidence that this event is related to TAC. Furthermore, we observed a 17-year-old boy developing ileus one month after the diagnosis of IMN and one single case of serious femoral head necrosis which was probably related to the exposure of the glucocorticoid in the TAC group.

### Secondary outcomes

**Figure 3.**
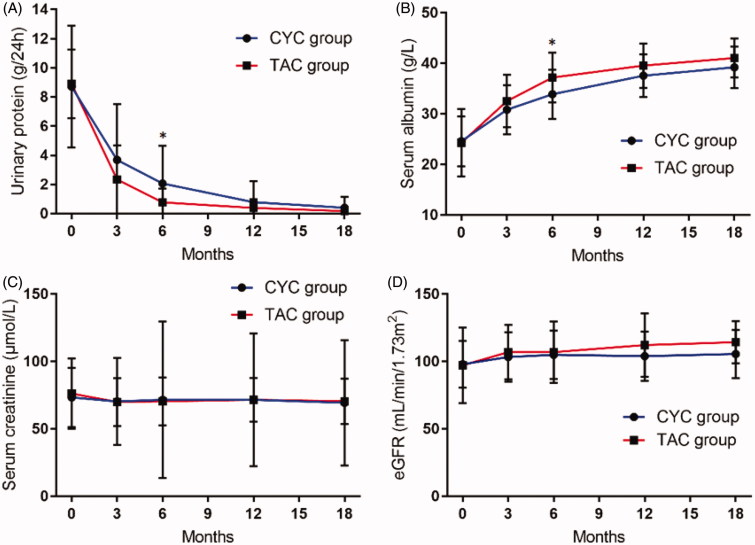
Evolution of the secondary end points in idiopathic membranous nephropathy patients treated with cyclophosphamde compared to tacrolimus. The line graphs for (A) 24 h urinary protein, (B) serum albumin, (C) serum creatinine, or (D) eGFR. CYC: cyclophosphamide; TAC: tacrolimus; eGFR: estimated glomerular filtration rate. *p < .05, CYC group *vs* TAC group (*t*-test).

As shown in Figure 3, from baseline to the end of 18-month, the mean of 24 h UP decreased from 8.68 to 0.41 g in the CYC group and from 8.89 to 0.17 g in the TAC group. The mean of serum albumin increased from 24.78 to 39.26 g/L in the CYC group and 24.54 to 41.09 g/L in the TAC group. Of note, 24 h UP (*p* = .001) and serum albumin (*p* = .009) in the TAC group obtained a better improvement than that in the CYC group at the follow-up visit of 6 months. There were no differences in the 24 h UP and serum albumin between both groups at any other follow-up visit (*p* > .05). No significant difference was found in Scr and eGFR between the two groups (*p* > .05).

## Discussion

Glucocorticoids in combination with alkylating agents or calcineurin inhibitors are the most two widely accepted protocols in treating patients with IMN. TAC is a more potent inhibitor of antigen-driven T cell activation, lymphocyte proliferation and cytokine production *in vitro* [11] and makes rare adverse effects in renal transplantation when compared to CYC [12–14]. Several studies report that TAC is effective in treating IMN patients whether monotherapy or combined with glucocorticoid [7–10]. However, all the previously mentioned studies had small sample sizes. In present study, we recruited 203 IMN patients who were prescribed either TAC or CYC with glucocorticoids for 18 months. We compared the effectiveness and safety profile of TAC plus glucocorticoid with combined CYC-glucocorticoid immunosuppressive regimen in two well defined cohorts of patients with IMN and NS.

Recently, researches from oriental cohorts all emphasized the critical role of proteinuria in IMN, which was a valuable prognostic indicator for both the clinician and patient. Attaining lower proteinuria predicted good renal survival [15–17]. Our data elucidated that, the incidence of remissions of the NS was significantly higher in the TAC group in the initial 3-month follow-up. The incidence rates of serious and non-serious adverse events were lower in the TAC group than the CYC group after the first 3-month follow-up. These results indicate the TAC plus glucocorticoid regimen is a promising alternative to CYC plus glucocorticoid as first-line immunosuppressive therapy in IMN. Beyond the inherent immunosuppressive effect, TAC can probably intensify the immunosuppressive action of glucocorticoids by potentiating glucocorticoid receptor affinity [18]. Our primary outcomes show that the incidence of remissions in the TAC group was significantly higher than the CYC group in the first 3 months. This may be one of the underlying mechanisms by which this regimen achieved satisfactory results in treating IMN. This result was comparable to previous studies [8,9]. Generally, achievement of remission in IMN patients could significantly reduce the risk of renal failure [19]. Our results showed that18-month course therapy of either CYC or TAC led to favorable sustained remission (93.7% *vs* 91.8%) and lower relapse rates (3.5% *vs* 4.9%). However, we observed that a small percentage of patients, especially in the TAC group relapsed within three years, but not during the 18-month follow-up period. Due to the severe lack of clinical data in most patients, we were unable to extend the follow-up period, resulting in a lower relapse rates in both groups.

As this was a retrospective study, the number of patients in the two treatment groups was different due to patients’ medication choices. The rates of ACEI/ARB use in both groups were low. The reasons for which patients in each group did not use might be that their blood pressures were too low to tolerate these medicines or other contraindications. There was no statistically significant difference in the incidence of adverse events. However, the higher rates of adverse events (any first serious adverse events or any first non-serious adverse events) were observed in the CYC group than the TAC group. The difference in incidence between the two groups became apparent after the first 3 months. 80 of the 88 serious events observed in the CYC group were considered likely or possibly treatment related, whereas 19 of the 22 serious events observed in the TAC group were considered as related to treatment.

Among treatment-related serious adverse events in the CYC group, there were 7 cases of myelotoxicity that were most probably because of a direct effect of the alkylating agent. Two cases of thromboembolic events were related to either the prothrombotic effect of glucocorticoids or hypoalbuminemia. The levels of serum albumin in these two patients were in the range of 25–26 g/L. The cases of new-onset diabetes mellitus and osteonecrosis were related to glucocorticoids therapy in the two groups. The frequency and severity of infections were also different between the two groups. All of the 11 serious adverse events in two groups, including 4 cases of cardiovascular events and three cases of infection were deemed to be unrelated to treatment. For instance, two cases of perirenal infections were caused by renal biopsy. One patient was diagnosed with ileus after admission. One fatal cardiovascular event was probably related to a previous history of cardiovascular disease. Among all the non-serious adverse events noted in two groups, there were 63 cases of infections, including respiratory tract (community-acquired and hospital-acquired), epifolliculitis, and herpes zoster. In addition, 13 cases of hyperglycemia, 40 cases of hepatotoxicity and 16 cases of new-onset tachycardia which all caused by glucocorticoids therapy were noted. Although the results of the present study did not support the hypothesis of non-inferiority in the TAC group in the aspect of adverse events, both the regimens had comparable short-term outcomes in the management of IMN and with a different spectrum of side-effects.

According to our secondary outcomes, the results indicate that significant differences in the levels of 24 h UP and serum albumin at the follow-up visit of 6 months. The underlying mechanism may be attributed to the protective effect of TAC on hepatocytes and its inhibitory effect on interleukin-6, which reduce the secretion of hepatic albumin [20]. During the 18-month period of treatment, Scr did not wave which is different to previously reported studies [9,21].

## Strengths and limitations

For the first time, we performed a retrospective study with long-term follow-up, and analyzed the safety profiles in two treatment groups. We firstly found that the combination of glucocorticoid and TAC had short-term benefits and long-term safety profile in patients with IMN. Our study had several limitations. This was a retrospective study, which might have retrospective bias. The number in the two groups was different due to patients’ choice of medication. There is a selection bias whereby young females are being treated with TAC and the older men are receiving CYC therapy. Nevertheless, the analyses were performed on the basis of predetermined study protocol and statistical plans. End point data were available from patient clinical records, which may have led to underestimation of adverse event rates. However, this underlying limitation applied to all patients and, therefore, is not anticipated to contribute to a systematic bias in support of one of the two treatment groups. Although the baseline characteristics did not significantly differ between the two groups, except in the age distribution, differences in diagnostic workup procedures or genetic background may have caused some residual confounding. Lastly, lack of anti-PLA2R assessment is one of the limitations in our research. It is regrettable for us that the measurement of anti-PLA2R was carried out in the mentioned hospital in recent years, so most patients lacked this laboratory testing results, which makes it difficult to make a decision on treatment and follow up. In conclusion, it is required for us to conduct RCT studies to evaluate the effectiveness and safety of TAC combined with glucocorticoid in treating IMN.

## Authors’ contributions

Honghong Zou performed the data collection, reviewed articles, and wrote the manuscript. Fang Jiang completed the data analysis and provided the second views during the manuscript preparation. Gaosi Xu designed the study and revised the manuscript. All the authors read and approved the final version of the manuscript.
